# Relative Centrifugal Force (RCF; *G*-Force) Affects the Distribution of TGF-β in PRF Membranes Produced Using Horizontal Centrifugation

**DOI:** 10.3390/ijms21207629

**Published:** 2020-10-15

**Authors:** Zahra Kargarpour, Jila Nasirzade, Layla Panahipour, Richard J. Miron, Reinhard Gruber

**Affiliations:** 1 Department of Oral Biology, Medical University of Vienna, 1090 Vienna, Austria; zahra.kargarpooresfahani@meduniwien.ac.at (Z.K.); jila.nasirzaderajiri@meduniwien.ac.at (J.N.); layla.panahipour@meduniwien.ac.at (L.P.); 2 Department of Periodontology, School of Dental Medicine, University of Bern, 3010 Bern, Switzerland; richard.miron@zmk.unibe.ch

**Keywords:** fibrin, platelet-rich fibrin, TGF-β, gravitational force, buffy coat, platelet-poor plasma

## Abstract

Solid platelet-rich fibrin (PRF) is produced with centrifugation tubes designed to accelerate clotting. Thus, activated platelets may accumulate within the fibrin-rich extracellular matrix even before centrifugation is initiated. It can thus be assumed that platelets and their growth factors such as transforming growth factor-β (TGF-β) are trapped within PRF independent of their relative centrifugal force (RCF), the gravitation or *g*-force. To test this assumption, we prepared PRF membranes with tubes where clotting is activated by a silicone-coated interior. Tubes underwent 210 *g*, 650 *g* and 1500 *g* for 12 min in a horizontal centrifuge. The respective PRF membranes, either in total or separated into a platelet-poor plasma and buffy coat fraction, were subjected to repeated freeze-thawing to prepare lysates. Gingival fibroblasts were exposed to the PRF lysates to provoke the expression of TGF-β target genes. We show here that the expression of interleukin 11 (IL11) and NADPH oxidase 4 (NOX4), and Smad2/3 signaling were similarly activated by all lysates when normalized to the size of the PRF membranes. Notably, platelet-poor plasma had significantly less TGF-β activity than the buffy coat fraction at both high-speed protocols. In contrast to our original assumption, the TGF-β activity in PRF lysates produced using horizontal centrifugation follows a gradient with increasing concentration from the platelet-poor plasma towards the buffy coat layer.

## 1. Introduction

Platelet-rich fibrin is produced by the centrifugation of venous blood that undergoes coagulation [1]. Platelet-rich fibrin (PRF) can then be used in various clinical indications, including ridge preservation after tooth extraction [2], to increase the width of keratinized mucosa around implants [3], or to prepare sticky bone prior to augmentation [4], as summarized recently [5]. PRF is also applied to support the healing of hard-to-heal ulcers [6]. However, coagulation of PRF within tubes is usually not spontaneously being enhanced by clotting activators. Unlike glass tubes, plastic tubes do not support rapid clotting; thus, plastic clot activator tubes usually contain siliceous substances and polyvinylpyrrolidone-like compounds to enhance the adherence and to facilitate rapid dissolution of silica into collected blood. PRF prepared by plastic tubes consequently accumulates the clot activators favoring faster clotting [7]. Rapid clotting and thus the impact of the clot activators was recently proposed to amend the current “gradient” theory of platelet distribution based on centrifugation protocols [8].

Previous findings [8] have started a debate about the various centrifugation protocols and their impact on the quality of the PRF, a quality that was mainly defined by the release of growth factors [9,10,11] and by provoking gene expression changes [12]. Centrifugation protocols were originally refined with the goal of an equal distribution and accumulation of platelets within the platelet-poor plasma and the buffy coat layer moving from the original L-PRF protocol (700 *g*, 12 min) towards a slower A-PRF protocol (210 *g*, 8–12 min) in fixed-angle centrifuges [9]. Recently, horizontal centrifugation was introduced, allowing a faster and sharper separation of the cells according to their density in H-PRF [13]. The impact of gravitation force (*g*-force) and time on the gradient of platelets and leucocytes in the tubes was measured in anticoagulated blood [14,15]. The impact of the clot activators was not considered here.

Blood coagulation is a rapid process that initiates during the blood draw and centrifugation process [16]. Coagulation is enhanced by the addition of a clot activator, such as silica. Activated clotting time is usually around 2 min [17]; thus, the coagulation is rapidly advancing even before the tubes are exposed to centrifugation forces. Usually, the recommended time between the blood drawing and start of centrifugation is 60–90 s; otherwise the size of the PRF membrane is negatively affected [18]. Noteworthy, it is not only the size of the PRF membrane that matters but attention has also been paid to the distribution of platelets within the PRF membranes [8], as platelets are rapidly activated during the coagulation cascade and thereby entrapped in the developing fibrin-rich extracellular matrix. Moreover, platelets release the content of their granules within a few seconds [19]. Considering that the fibrin-rich extracellular matrix can adsorb at least part of the growth factors from the granules, it is possible that their distribution in PRF is not significantly affected by various centrifugation protocols.

Transforming growth factor-β (TGF-β) is stored in the alpha granules of platelets and immediately released upon activation of the coagulation cascade [19]. TGF-β can then adsorb to the heparin-binding domain of fibrin (ogen) [20]. Thus, in blood collection tubes of accelerated clotting, it is possible that TGF-β adsorbs to the developing blood clot even before centrifugation is initiated and continues thereafter. Fibronectin [21] and vitronectin [22], both being part of the blood clot [23], bind TGF-β. In support of these molecular principles, immunostainings of PRF revealed that TGF-β1 is present throughout the matrix, with an accumulation on the distal surface from PRF clots produced using fixed-angle centrifuges [24], refining studies on the release of TGF-β into the supernatant of PRF [9,10]. Taken together, it might be hypothesized that not only the total TGF-β activity in the PRF membranes, but also the distribution of TGF-β, are hardly affected by the centrifugation protocol.

Considering that it is usually the entire PRF membrane, independent of the centrifugation protocol that is used in a clinical setting, the amount of TGF-β released from the PRF membranes is typically detected by immunoassays. It was only recently that the TGF-β activity in liquid PRF was determined by a bioassay [25]. Moreover, no attempts have previously been made to investigate the distribution of TGF-β activity inside the PRF membranes following various centrifugation protocols. Also, the TGF-β activity released upon fibrinolysis by plasmin (EC 3.4.21.7) has not been investigated so far [26]. Therefore, the aim of the present study was to compare the TGF-β activity in the lysates of PRF membranes produced by horizontal centrifugation using tubes with clot activators at different *g*-forces. We report here that the total TGF-β activity is similar among the PRF membranes produced using different protocols; however, despite the proposed binding of TGF-β to the fibrin-rich matrix, there is a clear gradient of TGF-β activity within the PRF membrane towards the buffy coat produced at higher centrifugation speeds.

## 2. Results

### 2.1. PRF Lysates Prepared at 210 g, 650 g and 1500 g Provoked an Equal Increase of TGF-β Target Genes

**Figure 1:**
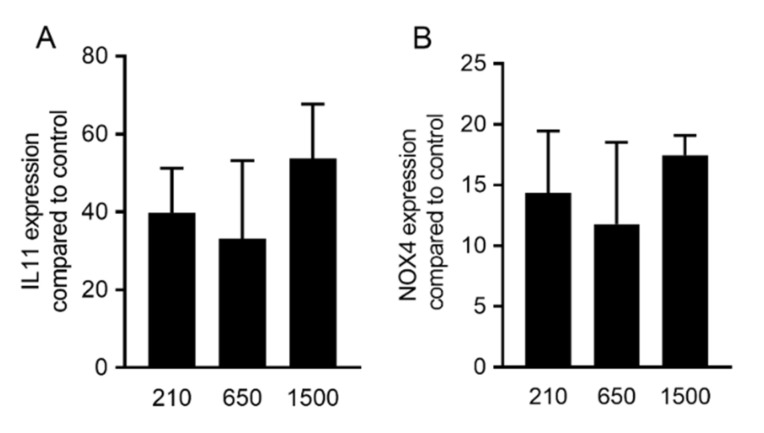
PRF lysates prepared at 210 *g*, 650 *g* and 1500 *g* provoked a robust increase of *TGF-β* target genes. Gingival fibroblast cells were exposed to 30% of PRF lysates prepared at different *g*-forces (210, 650 and 1500 *g*). Analysis of gene expression changes revealed that *IL11* (**A**) and *NOX4* (**B**) genes were upregulated in the presence of all variants of PRF lysates. Statistical analysis showed no significant differences (*p* > 0.05) between different variants of PRF lysates. These observations suggest that PRF lysates can activate the transforming growth factor-β (TGF-β) signaling pathway, regardless of the *g*-force applied. Statistical analysis was based on an ANOVA (*n* = 3).

To evaluate the effect of different *g*-forces on activation of TGF-β signaling, oral fibroblast cells were exposed to 30% of the PRF lysates prepared at different *g*-forces but normalized to the length of the PRF membrane (Table 1). Analysis of gene expression changes revealed that *IL11* and *NOX4* genes were upregulated in the presence of all variants of PRF lysates. Statistical analysis showed that the same increase of *IL11* and *NOX4* expression is achieved by 210 *g*, 650 *g* and 1500 *g* (*p* = 0.35, *p* = 0.27, respectively; Figure 1). These observations suggest that PRF lysates produced upon freeze-thawing and sonication, when normalized to the original length of the PRF membrane, can activate the TGF-β signaling pathway regardless of the *g*-force applied.

### 2.2. PRF Lysates Prepared at 210 g, 650 g and 1500 g Caused an Equal Production of IL11 Protein

**Figure 2:**
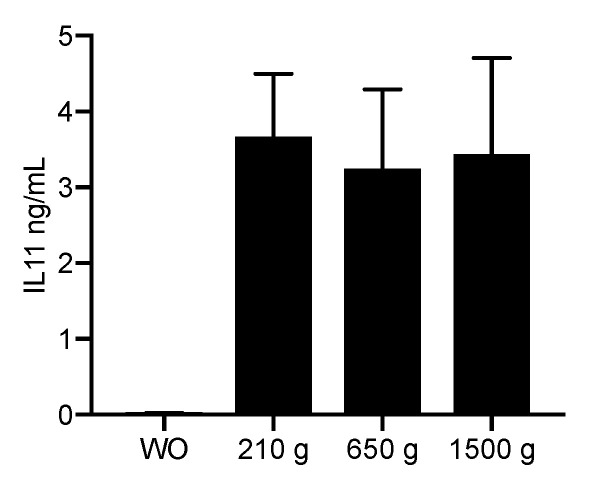
PRF lysates prepared at 210 *g*, 650 *g* and 1500 *g* accumulate IL11 in the cell supernatant. Gingival fibroblasts were exposed to 30% of PRF lysates prepared at 210 *g*, 650 *g* and 1500 *g*. The levels of IL11 in the supernatant of the fibroblasts are presented in ng/mL. Bar graphs show the means and standard deviation of four independent experiments. Statistical analysis was based on an ANOVA.

To confirm the findings obtained by gene expression analysis, we measured the levels of IL11 protein in the supernatant of gingival fibroblasts. In agreement with the strong increase of IL11 transcripts, all the normalized PRF lysates prepared at 210 *g*, 650 *g* and 1500 *g* had a substantial impact on the production of IL11 by gingival fibroblasts on the protein level compared to unstimulated controls (Figure 2). Importantly, the amount of IL11 production was strongly induced by all PRF protocols (all *p* < 0.001), but independent of the centrifugation protocol used to prepare the PRF membranes (all *p*-values around 0.9).

### 2.3. Expression Changes Were Blocked in the Presence of the TGF-β Receptor Type I Kinase Inhibitor

**Figure 3:**
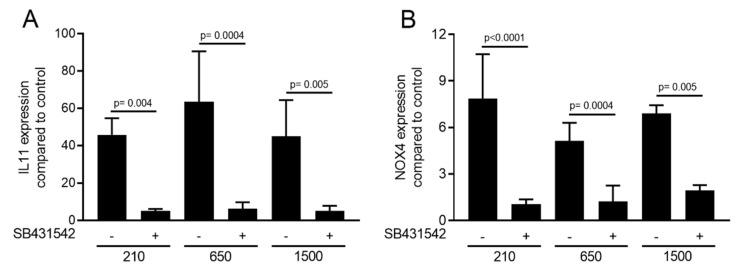
Expression changes were blocked in the presence of the TGF-β receptor type I kinase inhibitor. Gingival fibroblasts were exposed to 30% of PRF lysates prepared at different *g*-forces with and without the TGF-β receptor 1 antagonist SB431542. Expression analysis of (**A**) *IL11* and (**B**) *NOX4* is expressed as x-fold increases compared to the basal levels of untreated cells. Bar graphs show the means and standard deviation of three independent experiments. Bar graphs show the means and standard deviation of three independent experiments. Statistical analysis was based on a paired *t*-test.

To confirm the involvement of TGF-β signaling to regulate the respective genes, the TGF-β receptor 1 inhibitor SB431542 (10 µM) was applied. We report here that SB431542 blocked the activity of PRF lysates prepared at 210 *g*, 650 *g* and 1500 *g* to increase the expression of *IL11* and *NOX4*. These observations support the notion that the effects of the PRF lysates prepared at 210 *g*, 650 *g* and 1500 *g* are similarly mediated via the TGF-β receptor (Figure 3).

### 2.4. PRF Lysates Prepared at 210 g, 650 g and 1500 g Provoked an Equal Activation of Smad2/3 Signaling

**Figure 4:**
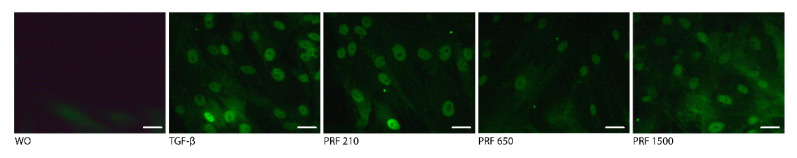
PRF lysates prepared at 210 *g*, 650 *g* and 1500 *g* provoked nuclear translocation of Smad2/3. Gingival fibroblasts were exposed for 1 h to 30% of the PRF lysates prepared at different *g*-forces. Immunofluorescence revealed the nuclear translocation of *Smad2/3* by all variants of PRF lysates compared to the untreated control. Scale bars are 100 µm. The Figure is representative for three independent experiments.

**Figure 5:**
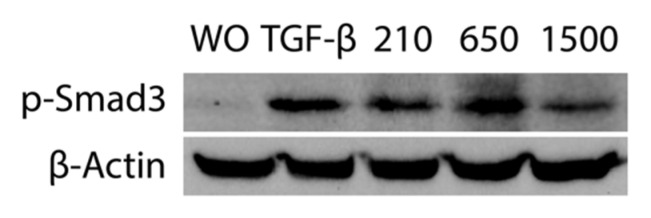
PRF lysates prepared at different *g*-forces activated phosphorylation of Smad3. Gingival fibroblasts were exposed to 30% of the PRF lysates prepared at 210 *g*, 650 *g* and 1500 *g* overnight. Western blot showed a strong increase of the basal Smad3 phosphorylation signal by all the PRF lysates. This Western blot is representative for three independent experiments.

In support of the activation of TGF-β receptor signaling [27], immunofluorescence and Western blot analysis were performed. Consistent with the proposed TGF-β activity, all variations of PRF lysates prepared at 210 *g*, 650 *g* and 1500 *g* provoked the nuclear translocation of Smad2/3 in gingival fibroblasts. Again, the magnitude of nuclear translocation of Smad2/3 was visibly similar among the three PRF protocols (Figure 4). Considering that phosphorylation of Smad3 is a prerequisite for its nuclear translocation, we showed that consistently all three separate protocols of PRF could provoke an increased phosphorylation of Smad3, and more importantly, with the same magnitude (Figure 5).

### 2.5. PPP Lysates Prepared at 1500 g Failed to Increase the Expression of TGF-β Target Genes

**Figure 6:**
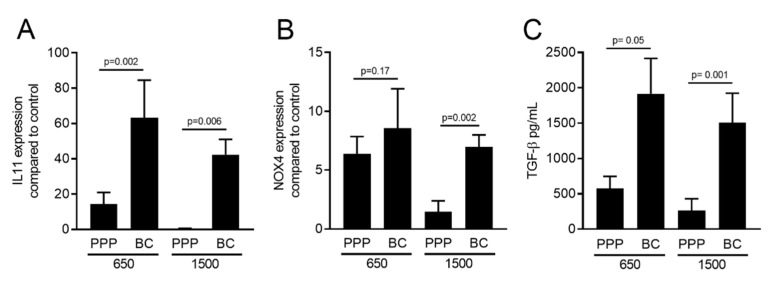
The buffy coat (BC) fraction has significantly more TGF-β activity compared to platelet-poor plasma (PPP) in PRF 1500 *g* and 650 *g*. Gingival fibroblasts were exposed to 30% of lysates from PPP and BC layers of PRF prepared at 650 *g* and 1500 *g*. Analysis of gene expression changes revealed that *IL11* (**A**) was upregulated significantly in the BC layer of PRF prepared at 1500 *g* and 650 *g*, and the *NOX4* (**B**) gene was upregulated in the presence of all variants of PRF lysates. Statistical analysis showed no significant differences (*p* > 0.05) between the PPP and BC layers in *NOX4* gene expression. These observations suggest that the BC layer has more TGF-β activity compared to the uppermost PPP layer (*n* = 6). Concentration of active TGF-β in the BC and PPP fractions of PRF prepared at 1500 *g* and 650 *g* was evaluated by immunoassay (**C**). Data represent the mean and standard deviation. TGF-β is indicated in pg/mL. Statistical analysis was conducted using ANOVA (*n* = 6).

**Figure 7:**
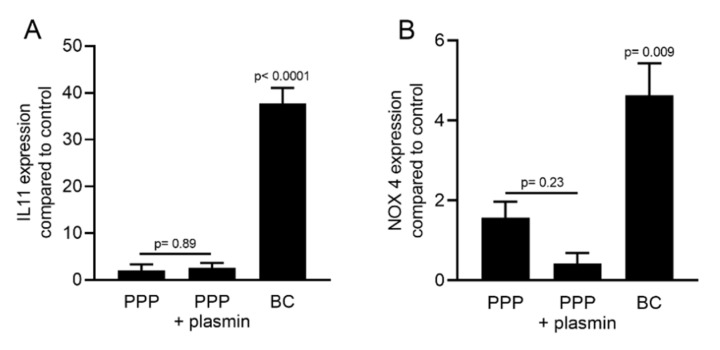
The plasmin-digested PPP fraction at 1500 *g* did not increase TGF-β target genes expression. Gingival fibroblasts were exposed to 30% of the BC and PPP fraction of PRF lysates prepared at 1500 *g* and the plasmin-digested PPP fraction. Gene expression analysis indicated that plasmin digestion is not sufficient for PPP to make changes in the expression of *IL11* (**A**) and *NOX4* (**B**). Data represent the mean and standard deviation of three independent experiments. Statistical analysis was performed using ANOVA (*n* = 3).

To gain insight into the distribution of TGF-β activity in different parts of the membranes, the PRF membranes prepared at 1500 *g* and 650 *g* were cut into two parts. One part was taken close to the red blood cell layer, representing the buffy coat (BC), and the upper layer was termed platelet-poor plasma (PPP). Gingival fibroblasts were exposed to 30% lysates of PPP or the BC fraction followed by *IL11* and *NOX4* gene expression analysis. The BC fraction of the PRF membranes produced at 1500 *g* and 650 *g* demonstrated a significantly better capacity to increase *IL11* and *NOX4* expression than the respective PPP fractions (Figure 6A,B). Noteworthy, the TGF-β activity was completely lacking in the PPP layer when prepared using the 1500 *g* protocol. Immunoassay confirmed that the amount of active TGF-β1 protein was significantly higher in the BC compared to the PPP fraction produced at either 1500 *g* or 650 *g* (Figure 6C). Plasmin-mediated fibrinolysis of the PPP layer produced with the 1500 *g* protocol failed to release TGF-β activity (Figure 7).

### 2.6. PPP Lysates Prepared at 1500 g Failed to Considerably Activate Phosphorylation of Smad3

**Figure 8:**
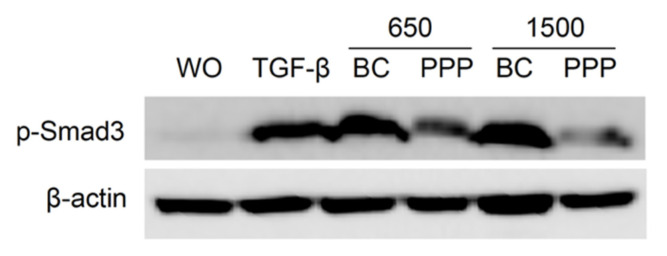
The BC fraction of PRF 650 *g* and 1500 *g* provoked phosphorylation of Smad3. Gingival fibroblasts were exposed to 30% of the BC and PPP fractions of PRF lysates prepared at 650 *g* and 1500 *g* for 16 h. Western blot showed a strong increase of the basal Smad3 phosphorylation signal by both BC fractions compared to the weak signal generated by PPP fractions.

In support of the activation of TGF-β receptor signaling mainly by BC and not PPP, phosphorylation of Smad3 was evaluated. Gingival fibroblasts were exposed to 30% of the BC and PPP fractions of PRF lysates prepared at 650 *g* and 1500 *g* for 16 h. Western blot indicated that BC fractions of PRF lysates prepared at 650 *g* and 1500 *g* provoked an increased phosphorylation of Smad3. In contrast, PPP fractions failed to generate a similar response (Figure 8).

## 3. Discussion

The present research is based on the concept that clot activator blood collection tubes simultaneously activate platelets and kick-start the formation of a fibrin-rich extracellular matrix. TGF-β, being rapidly released from platelets, is then bonded to the fibrin-rich extracellular matrix and thereby escapes from the *g*-force produced by the centrifugation forces. Thus, when using the clot activator blood collection tubes, the TGF-β activity in the PRF membranes is theoretically independent of *g*-force. We report here that the TGF-β activity of normalized PRF lysates produced at 210 *g*, 650 *g* and 1500 *g* for 12 min in a horizontal centrifuge is indeed rather similar. Considering that the size of the PRF membranes increases with increasing *g*-forces, the total TGF-β activity also has the potential to increase. With increasing *g*-forces, however, the PPP fraction gradually loses its TGF-β activity. This finding is important because it shows that even in clot activator blood collection tubes, the TGF-β activity in PRF membranes produced at high *g*-forces follows a gradient.

If we relate our findings to those observed with liquid PRF produced at 700 *g* for 8 min, the PPP fraction provokes less activation of *IL11* and *NOX4* genes compared to the respective buffy coat layer [25]. We now show that with solid PRF produced at 650 *g* for 12 min, the PPP fraction provoked less activation of *IL11* and *NOX4* genes than the respective buffy coat layer. We can thus conclude that in liquid but also in solid PRF protocols using a horizontal centrifuge, the TGF-β activity follows a gradient. Solid PPP produced at 1500 *g* for 12 min is even almost devoid of TGF-β activity. This observation was originally surprising as we hypothesized that platelets would rapidly aggregate on polymerizing fibrin and the TGF-β activity is retained in the PPP even at 1500 *g* [28]. Nevertheless, in clot activator blood collection tubes, there is a gradient of TGF-β activity being high in the BC but almost null in the PPP fraction produced with high-speed centrifugation.

It is noteworthy to translate our findings obtained with horizontal centrifuges to those observed with fixed-angle centrifuges when preparing solid PRF. Fibrin polymerization is triggered by the contact between coagulation factor XII and a procoagulation glass surface; thus, in fixed-angle centrifuges, platelets are pressed to and accumulate on the distal side of the tube [8]. Likewise, in fixed-angle centrifuges, TGF-β1 is relatively evenly distributed within the PRF matrix from the upper PPP layer down to the BC layer [24]. This distribution along the back walls produced using fixed-angle centrifuges can be explained by TGF-β1 being rapidly released from platelets hitting the distal wall [19], and being then adsorbed by fibrin (ogen) [20], fibronectin [21] and vitronectin [22] of the blood clot [23]. The distribution of TGF-β in solid PRF produced by horizontal and fixed-angle centrifuges might thus be different.

We can speculate that at high-speed protocols, platelets accumulate in the buffy coat fraction where they release the content of their granules, including TGF-β that adsorbs to the fibrin-rich extracellular matrix. This concept is supported by the general observation that TGF-β1 is released over days from solid PRF membranes [9,10] that undergo spontaneous fibrinolysis. TGF-β being released within days can theoretically originate from the platelets or leucocytes that continue producing TGF-β; this theory is unlikely as platelets have no nucleus, so they cannot produce TGF-β like megakaryocytes [29]. Monocytes and neutrophils can produce TGF-β [30] but monocytes have to be activated and neutrophils have a limited life span; thus, both cell types are not privileged to release considerable amounts of TGF-β over days. It is presumably the intrinsic activation of fibrinolysis that causes the release of TGF-β from PRF membranes.

Fibrinolysis of PRF clots can be activated by the microbiome, including *Staphylococcus aureus*, *Streptococcus pyogenes* and *Candida albicans* [31,32]. The periodontal pathogen *Porphyromonas gingivalis* also supports fibrinolysis [33]. Practically, however, the PRF membranes are produced ex vivo, avoiding bacteria and fungi. Thus, the PRF membrane is probably protected from the fibrinolytic activity of the microbiome, and the PRF membrane even possesses antibacterial effects, at least for *P. gingivalis* [34]. To simulate fibrinolysis, though, we have digested the PPP membranes with plasmin. PPP lysates failed to enhance the expression of *IL11* and *NOX4* in fibroblasts. Thus, assuming that TGF-β theoretically remains bound to PPP, it is not liberated by plasmin-mediated fibrinolysis. Future studies should reveal if PPP holds back any TGF-β activity and how cells immigrating in PRF membranes can liberate the TGF-β from the extracellular matrix [20,21,22].

The present study has a number of limitations. First, regarding the debates on the impact of cell distribution on the activity of different parts of PRF, we only considered PRF prepared at 1500 *g* and 650 *g*. This exclusion was because the membrane from PRF prepared at 210 *g* was not big enough to be sliced into two considerable parts and studied. Second, the screening for target genes is based on a selection of two marker genes which are upregulated upon TGF-β receptor activation, mainly based on our previous research with PRF [10] and acid bone lysates [35]. Therefore, other genes that are responsive to growth factors other than TGF-β might have been oversighted. Furthermore, gingival fibroblasts represent one out of many possible target cells for PRF and it is likely that the beneficial effect of PRF in vivo requires the crosstalk between multiple cell types. The robust TGF-β activation might only be relevant for mesenchymal cells while other cell types require the activation by other components apart from growth factors.

Considering these limitations, we can nevertheless conclude that the TGF-β activity, when normalized to the size of the PRF membranes, is rather equal among the three protocols produced using horizontal centrifugation. Since the size of the PRF membrane increases with speed, the total TGF-β also increases at high-speed centrifugation. We further have to conclude that in horizontal centrifuges, speed affects the distribution of TGF-β in the PRF clot, with almost no TGF-β activity in the PPP fraction.

## 4. Materials and Methods

### 4.1. Cell Culture

Human gingiva was harvested from extracted wisdom teeth from patients who had given informed and written consent. An approval was obtained from the Ethics Committee of the Medical University of Vienna (EK NR 631/2007). A total of three strains of fibroblasts were established by explant cultures and fewer than 10 passages were used for the experiments. Gingival fibroblasts were grown and supplemented with 1% antibiotics (Sigma-Aldrich, St. Louis, MO, USA.) and 10% fetal calf serum (Bio&Sell GmbH, Nuremberg, Germany). The cells were seeded at 30,000 cells/cm^2^ onto culture dishes one day prior to stimulation. Cells were exposed to 30% lysates of PRF prepared at 210 *g*, 650 *g* and 1500 *g* for another 24 h under standard conditions at 37 °C, 5% CO_2_ and 95% humidity. To examine the influence of TGF-β signaling, the inhibitor of TGF-β receptor type I kinase, SB431542 (Calbiochem, Merck, Billerica, MA, USA), was used at 10 µM.

### 4.2. Preparation of PRF Lysates

**Figure 9:**
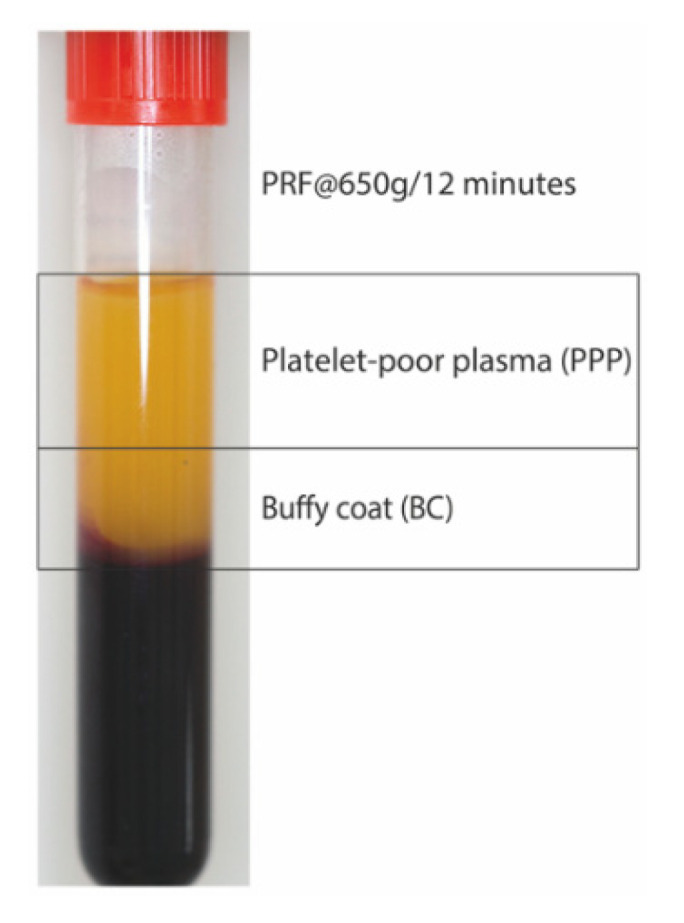
PRF prepared at 650 *g* in a horizontal centrifuge. The yellow PRF clot was compressed between two layers of dry gauze. Either the total PRF membrane or the PRF membrane being cut into two pieces representing the buffy coat (BC) and the platelet-poor plasma (PPP) was used to prepare the lysates.

PRF was prepared after the approval of the Ethics Committee of the Medical University of Vienna (1644/2018) and volunteers signed informed consent. All experiments were performed in accordance with relevant guidelines and regulations. For preparation of PRF lysates, venous blood was collected at the University Clinic of Dentistry from six healthy volunteers in glass-coated tubes (BD vacutainer, BD368045, Plymouth, UK) and centrifuged at 210 *g*, 650 *g* (Figure 9) and 1500 *g* for 12 min (Z 306, Hermle, Universal Centrifuge, Wehingen, Germany) with universal swing-out rotors (146 mm at the max). The PRF clot was separated from the remaining red thrombus and compressed between two layers of dry gauze. Each PRF membrane was transferred into serum-free medium (1 cm PRF/mL) and exposed to two cycles of freeze-thawing and sonication (Sonopuls 2000.2, BANDELIN electronic, Berlin, Germany). The lysate was subjected to centrifugation at 15,000 *g* for 10 min and the respective supernatant was filtered sterile and stored at −20 °C before the in vitro analysis [36,37,38]. Exclusively, PRF membrane prepared at 1500 *g* and 650 *g* (Figure 9) were also cut into two pieces closed to the border (buffy coat; BC) and in the most upper part (platelet-poor plasma; PPP) of the membranes. For indicated experiments, the 1500 *g* PPP membrane was incubated with 5 nM plasmin (Sigma-Aldrich, St. Louis, MO, USA.) in Tris-HCl 50 mM (pH = 7.4) [39]. Following fibrinolysis at 37 °C for 4 h, the reaction was terminated by heating at 65 °C for 15 min before centrifugation and sterile filtration was performed.

### 4.3. RT-qPCR Analysis and Immunoassay

For reverse transcription quantitative real-time PCR (RT-qPCR), total RNA was isolated with the ExtractMe total RNA kit (Blirt S.A., Gdańsk, Poland). Reverse transcription was performed with cDNA synthesis kit (LabQ, Labconsulting, Vienna, Austria). Polymerase chain reaction was performed with the RT-PCR Kit (LabQ, Labconsulting, Vienna, Austria). Intron-spanning primers were designed based on the algorithm of the Universal ProbeLibrary Assay Design Center (www.lifescience.roche.com) and primer sequences were hGAPDH-F: aag cca cat cgc tca gac ac; hGAPDH-R: gcc caa tac gac caa atc c.; hNOX4a-F tct tgg ctt acc tcc gag ga; hNOX4a-R: ctc ctg gtt ctc ctg ctt gg and hIL11 (qHsaCEP0049951; Bio-Rad Laboratories, Inc., Hercules, CA). Amplification was monitored on the CFX Connect^TM^ Real-Time PCR Detection System (Bio-Rad Laboratories, CA, USA). The mRNA levels were calculated by normalizing to the housekeeping gene GAPDH using the ΔΔCt method. The immunoassay for human IL11 (DY218, R&D Systems, Minneapolis, MN, USA) was performed with the supernatant of gingival fibroblasts exposed to PRF lysates at different *g*-forces after 24 h.

### 4.4. Immunofluorescence

The immunofluorescent analysis of Smad2/3 was performed in gingival fibroblasts plated onto Millicell^®^ EZ slides (Merck KGaA, Darmstadt, Germany) at 15,000 cells/cm^2^. Cells were exposed to 30% of PRF prepared at 210 *g*, 650 *g* and 1500 *g* overnight. In indicated experiments, cells were exposed to 30% of BC and PPP fractions of PRF prepared at 650 *g* and 1500 *g*. The cells were fixed with 4% paraformaldehyde, blocked with 1% bovine serum albumin and permeabilized with 0.3% Triton. We used rabbit anti-Smad2/3 antibody (D7G7 XP^®^) at 4 °C overnight. Detection was with the goat anti-rabbit Alexa 488 secondary antibody (CS-4412, 1:1000, Cell Signaling Technology). Images were captured under a fluorescent microscope with a single filter block 455 nm (Oxion fluorescence, Euromex, Arnheim, The Netherlands).

### 4.5. Western Blot

Gingival fibroblasts were seeded at 30,000 cells/cm^2^ into 6-well plates. The following day, the medium was changed to serum-free for 8 h following stimulation for 16 h with 30% of PRF lysates prepared at 210 *g*, 650 *g* and 1500 *g* or the PPP and BC fractions prepared at 650 *g* and 1500 *g*. Extracts containing SDS buffer with protease and phosphatase inhibitors (cOmplete ULTRA Tablets and PhosSTOP; Roche, Mannheim, Germany) were separated by SDS-PAGE and transferred onto nitrocellulose membranes (Whatman, GE Healthcare, General Electric Company, Fairfield, CT, USA). Membranes were blocked, and the binding of the first antibody rabbit phospho S423 + S425 (p-Smad3) antibody (EP823Y, Abcam, Cambridge, UK) was detected with the second antibody labeled with HRP (CS-7074, anti-rabbit IgG, 1:10,000, Cell Signaling Technology), respectively. After exposure to the Clarity Western ECL Substrate (Bio-Rad Laboratories, Inc., Hercules, CA, USA) chemiluminescence signals were visualized with the ChemiDoc imaging system (Bio-Rad Laboratories).

### 4.6. Statistical Analysis

All experiments were performed three to five times. Bars show the mean and standard deviation of the cumulative data from the means of independent experiments. Statistical analysis of the *IL11* and *NOX4* expression and immunoassay was performed with ANOVA and post-hoc Fischer’s LSD test for multiple comparisons. An ANOVA and post-hoc Fischer’s LSD test for multiple comparisons compared all groups with each other. Analyses were performed using Prism v8 (GraphPad Software, La Jolla, CA, USA). Significance was set at *p* < 0.05.

## Figures and Tables

**Table 1:** The size of platelet-rich fibrin (PRF) membranes prepared at different *g*-forces are listed below in cm.

	210 *g* (cm)	650 *g* (cm)	1500 *g* (cm)
Donor 1	1.5	3.0	4.0
Donor 2	2.0	2.5	3.5
Donor 3	2.0	3.0	4.0
Donor 4	2.0	3.0	3.5
